# The performance of the WHO COVID-19 severity classification, COVID-GRAM, VACO Index, 4C Mortality, and CURB-65 prognostic scores in hospitalized COVID-19 patients: data on 4014 patients from a tertiary center registry

**DOI:** 10.3325/cmj.2023.64.13

**Published:** 2023-02

**Authors:** Marko Lucijanić, Nevenka Piskač Živković, Tanja Režić, Ivan Durlen, Josip Stojić, Ivana Jurin, Sara Šakota, Dora Filipović, Iva Kurjaković, Ana Jordan, Nikolina Bušić, Josipa Pavić, Ivica Lukšić, Bruno Baršić

**Affiliations:** 1 Hematology Department, Dubrava University Hospital, Zagreb, Croatia; 2 Primary Respiratory and Intensive Care Center, Dubrava University Hospital, Zagreb, Croatia; 3 University of Zagreb, School of Medicine, Zagreb, Croatia; 4 Pulmonology Department, Dubrava University Hospital, Zagreb, Croatia; 5 Endocrinology Department, Dubrava University Hospital, Zagreb, Croatia; 6 Nephrology Department, Dubrava University Hospital, Zagreb, Croatia; 7 Department of Emergency and Intensive Care Medicine, Dubrava University Hospital, Zagreb, Croatia; 8 Cardiology Department, Dubrava University Hospital, Zagreb, Croatia; 9 Department of Medical Biochemistry and Hematology Laboratory, Livno General County Hospital, Livno, Bosnia and Herzegovina; 10 Department of Maxillofacial Surgery, Dubrava University Hospital, Zagreb, Croatia

## Abstract

**Aim:**

To evaluate the predictive properties of several common prognostic scores regarding survival outcomes in hospitalized COVID-19 patients.

**Methods:**

We retrospectively reviewed the medical records of 4014 consecutive COVID-19 patients hospitalized in our tertiary level institution from March 2020 to March 2021. Prognostic properties of the WHO COVID-19 severity classification, COVID-GRAM, Veterans Health Administration COVID-19 (VACO) Index, 4C Mortality Score, and CURB-65 score regarding 30-day mortality, in-hospital mortality, presence of severe or critical disease on admission, need for an intensive care unit treatment, and mechanical ventilation during hospitalization were evaluated.

**Results:**

All of the investigated prognostic scores significantly distinguished between groups of patients with different 30-day mortality. The CURB-65 and 4C Mortality Score had the best prognostic properties for prediction of 30-day mortality (area under the curve [AUC] 0.761 for both) and in-hospital mortality (AUC 0.757 and 0.762, respectively). The 4C Mortality Score and COVID-GRAM best predicted the presence of severe or critical disease (AUC 0.785 and 0.717, respectively). In the multivariate analysis evaluating 30-day mortality, all scores mutually independently provided additional prognostic information, except the VACO Index, whose prognostic properties were redundant.

**Conclusion:**

Complex prognostic scores based on many parameters and comorbid conditions did not have better prognostic properties regarding survival outcomes than a simple CURB-65 prognostic score. CURB-65 also provides the largest number of prognostic categories (five), allowing more precise risk stratification than other prognostic scores.

Before the start of vaccination, COVID-19 was characterized by a high proportion of patients developing respiratory insufficiency and requiring hospital admission. Vaccination successfully reduced the number of severe/critical patients and resulted in an improved clinical course of the disease (1). Nevertheless, vaccine hesitancy and waning immunity remain obstacles to a successful vaccination program (2,3). In patients developing severe/critical COVID-19, progressive respiratory deterioration is usually accompanied by a disorder in a number of other affected organ systems, such as the circulatory, hepatobiliary, and central nervous systems (4-7). The presence of comorbidities (8) and the severity of inflammatory process (9) are important predictors of unfavorable outcomes. A number of prognostic systems developed before and during the pandemic have been investigated in COVID-19 patients (10). These scores estimate respiratory, inflammatory, and comorbidity status, and perform differently in various cohorts of patients. Due to uncertainties regarding the prognostication of hospitalized patients with severe or critical presentation of COVID-19, we aimed to evaluate several common prognostic scores in patients from our tertiary level institution.

## Patients and methods

We retrospectively evaluated the electronic and paper medical records of 4014 consecutive COVID-19 patients admitted to Dubrava University Hospital, a tertiary-level institution, from March 2020 to March 2021. Baseline clinical and laboratory data as well as clinical outcomes of patients were recorded as a part of a Hospital Registry Project. All patients were of white race. All patients had positive polymerase chain reaction or antigen COVID-19 test before hospital admission. During hospital admission, patients were treated according to contemporary guidelines with various exposures to low molecular weight heparin (LMWH), corticosteroids, and remdesivir. The study was approved by the Institutional Review Board of Dubrava University Hospital (2021/2503-04).

Disease severity on admission was determined according to the World Health Organization (WHO) as mild, moderate, severe, or critical (11). In addition to WHO COVID-19 severity classification, the following prognostic scores were evaluated:

1) The COVID-GRAM score (12) was developed to evaluate the risk of critical illness among hospitalized patients with presumed COVID-19. It is based on the presence of x-ray abnormalities, age, hemoptysis, dyspnea, unconsciousness, number of comorbidities, cancer history, neutrophil-to-lymphocyte ratio, lactate dehydrogenase, and direct bilirubin. Patients are stratified into three risk categories.

2) The Veterans Health Administration COVID-19 (VACO) Index (13) was originally developed to evaluate 30-day mortality in potential COVID-19 patients. It is based on demographic parameters (age, sex) and comorbidities. The score incorporates no actual disease severity. Patients are stratified into four risk categories.

3) The 4C Mortality Score (14) was developed to evaluate the in-hospital mortality of COVID-19 patients. It is based on age, sex, the number of comorbidities, respiratory rate, peripheral oxygen saturation on room air, the Glasgow Coma Score, urea, and C-reactive protein (CRP). Patients are stratified into four risk categories.

4) The CURB-65 score (15) was originally developed to evaluate the mortality of community-acquired pneumonia patients. It is based on confusion, urea, respiratory rate, blood pressure, and age. Patients are stratified into five risk categories.

### Statistical analysis

The normality of distribution of numerical variables was assessed with a Kolmogorov-Smirnov test. Numerical variables are presented as median and interquartile range (IQR) and were compared between the groups with a Mann-Whitney U test. Categorical variables are presented as frequencies and percentages and were compared between the groups with a Χ^2^ test. The receiver operating characteristic (ROC) curve analysis was used to assess the predictive properties of prognostic scores regarding clinical outcomes of interest (30-day mortality, in-hospital mortality, presence of severe or critical disease on admission, need for an intensive care unit, and mechanical ventilation during hospitalization). Kaplan-Meier survival analysis was used, and survival curves were compared between the groups with the Cox-Mantel version of the log-rank test (16,17). The Cox regression analysis was used for multivariate survival analysis. *P* values <0.05 were considered statistically significant. All analyses were performed with the MedCalc Statistical Software, version 20.110 (MedCalc Software Ltd, Ostend, Belgium).

## Results

### Patients’ characteristics and COVID-19 prognostic scores

The study enrolled 4014 patients (2256, or 56.2% men) admitted to hospital for acute COVID-19. The median age was 74 years, IQR (64-82). The median Charlson comorbidity index was 4, IQR (3-6). At hospital admission, 3531 (88%) patients had pneumonia, 3265 (81.3%) required oxygen supplementation therapy, and 3359 (83.7%) presented with severe or critical COVID-19 symptoms. A total of 913 (22.7%) required intensive care unit treatment, 771 (19.2%) required high-flow oxygen therapy, and 675 (16.8%) required mechanical ventilation. A total of 1428 (35.6%) patients died.

**Figure 1:**
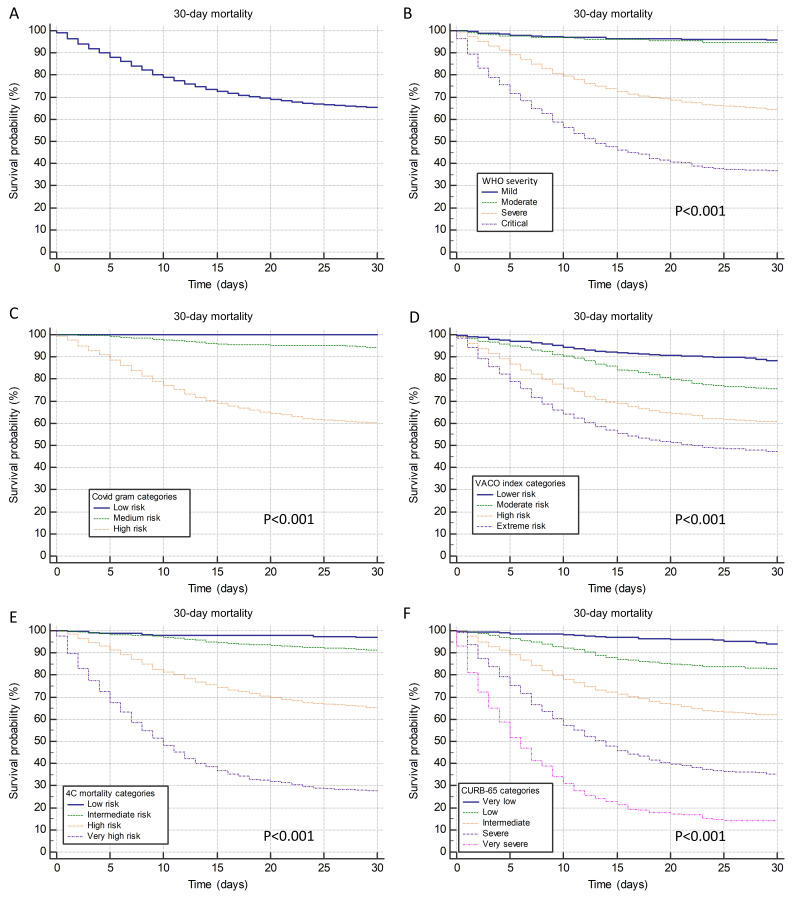
Thirty-day mortality curves for (**A)** the overall cohort and curves stratified by prognostic categories of (**B)** World Health Organization (WHO) COVID-19 severity classification, (**C)** COVID-GRAM, (**D)** The Veterans Health Administration COVID-19 Index (VACO) index, (**E)** 4C Mortality Score, and (**F)** CURB-65 score.

Patients’ characteristics and risk scores categories stratified according to in-hospital mortality are shown in Table 1. Thirty-day mortality curves for the entire cohort and stratified by the categories of the WHO severity, COVID-GRAM, VACO Index, 4C Mortality, and CURB-65 prognostic scores are shown in Figure 1A-F. All of the investigated prognostic scores significantly distinguished between groups of patients with different prognosis (overall *P* < 0.001 for all analyses).

**Table 1:** Patients’ characteristics and COVID-19 prognostic scores stratified according to in-hospital mortality*

	Overall (N = 4014)	Survival (n = 2586)	Death (n = 1428)	*P*
**Age** (years)	74 (64-82)	70 (60-80)	79 (72-85)	<0.001
**Male sex**	2256 (56.2)	1446 (55.9)	810 (56.7)	0.622
**Day of disease on admission**	5 (1-9)	5 (1-10)	4 (1-8)	<0.001
**Charlson comorbidity index**	4 (3-6)	4 (2-5)	5 (4-7)	<0.001
**Modified Early Warning Score**	2 (1-4)	2 (1-3)	3 (2-5)	<0.001
**In-hospital mortality**	1428 (35.6)	-	-	-
**30-day mortality**	1388 (34.6)	23 (0.9)	1365 (95.6)	-
**Intensive care unit**	913 (22.7)	240 (9.3)	673 (47.1)	<0.001
**Mechanical ventilation**	675 (16.8)	74 (2.9)	601 (42.1)	<0.001
**The World Health Organization severity categories**				<0.001
mild symptoms	449 (11.2)	433 (16.7)	16 (1.1)	<0.001
moderate symptoms	206 (5.1)	197 (7.6)	9 (0.6)	<0.001
severe symptoms	2761 (68.8)	1744 (67.4)	1017 (71.2)	0.013
critical symptoms	598 (14.9)	212 (8.2)	386 (27)	<0.001
**4C Mortality Score**	11 (8-14)	9 (7-12)	14 (12-16)	<0.001
**4C Mortality Score categories**				<0.001
low risk	227 (5.9)	220 (8.8)	7 (0.5)	<0.001
intermediate risk	900 (23.3)	814 (32.7)	86 (6.3)	<0.001
high risk	1943 (50.3)	1251 (50.2)	692 (50.4)	0.878
very high risk	795 (20.6)	208 (8.3)	587 (42.8)	<0.001
**COVID-GRAM score**	171 (145-203)	157 (136-182)	200 (173-234)	<0.001
**COVID-GRAM risk**	0.74 (0.47-0.93)	0.61 (0.38-0.83)	0.92 (0.76-0.98)	<0.001
**COVID-GRAM categories**				<0.001
low risk	4 (0.2)	4 (0.2)	0 (0)	0.147
medium risk	485 (19.3)	455 (27.7)	30 (3.5)	<0.001
high risk	2020 (80.5)	1185 (72.1)	835 (96.5)	<0.001
**The Veterans Health Administration COVID-19 (*VACO*) index score**	0.18 (0.09-0.27)	0.15 (0.07-0.21)	0.23 (0.16-0.32)	<0.001
**VACO index categories**				<0.001
lower risk	954 (23.9)	835 (32.4)	119 (8.4)	<0.001
moderate risk	756 (18.9)	558 (21.7)	198 (13.9)	<0.001
high risk	851 (21.3)	510 (19.8)	341 (23.9)	0.002
extreme risk	1438 (36)	671 (26.1)	767 (53.8)	<0.001
**CURB-65 score**	2 (1-2)	1 (1-2)	2 (2-3)	<0.001
**CURB-65 categories**				<0.001
very low risk	573 (14.7)	534 (21.2)	39 (2.8)	<0.001
low risk	1018 (26)	830 (32.9)	188 (13.6)	<0.001
intermediate risk	1520 (38.9)	925 (36.7)	595 (42.9)	<0.001
severe risk	607 (15.5)	207 (8.2)	400 (28.8)	<0.001
very severe risk	191 (4.9)	26 (1)	165 (11.9)	<0.001

*Data are presented as median (interquartile range) or frequency (percent).

The WHO COVID-19 severity classification did not differentiate mild from moderate patients, but it significantly differentiated the patients with severe and critical symptoms from each other and from lower-risk groups (Figure 1B, overall *P* < 0.001). Thirty-day mortality rates were 4.2%, 5.3%, 35.5%, and 63.4% for mild, moderate, severe, and critical groups, respectively.

The COVID-GRAM identified a low-risk group of patients in whom did no events occurred and who could not be statistically compared with the other groups. Patients belonging to the high-risk group significantly differed in survival from the medium-risk group (Figure 1C, overall *P* < 0.001). Thirty-day mortality rates were 0%, 5.8%, and 33.2% for low-, medium-, and high-risk groups, respectively.

The VACO Index distinguished between four groups of patients with significantly different prognosis (Figure 1D, overall *P* < 0.001). Thirty-day mortality rates were 11.6%, 24.5%, 39%, and 58.6% for lower-, moderate-, high-, and extreme-risk groups, respectively.

The 4C Mortality Score distinguished between four groups of patients with significantly different prognosis (Figure 1E, overall *P* < 0.001). Thirty-day mortality rates were 4.1%, 8.8%, 34.4%, and 72.3% for low, intermediate, high, and very high risk groups, respectively.

The CURB-65 distinguished between five groups of patients with significantly different prognosis (Figure 1F, overall *P* < 0.001). Thirty-day mortality rates were 5.9%, 7.2%, 38.1%, 64.9%, and 85.9% for very low-, low-, intermediate-, high-, and very high-risk groups, respectively.

### Comparison of prognostic properties of COVID-19 prognostic scores regarding different clinical outcomes in the entire cohort

The CURB-65 and 4C Mortality Score demonstrated an overall best performance in correctly classifying death-related outcomes. They had the best AUC values of similar magnitude, which were significantly better than those of the other indices for 30-day mortality (AUC 0.761 and 0.761 for CURB-65 and 4C Mortality Score, respectively) and for in-hospital mortality (AUC 0.757 and 0.762 for CURB-65 and 4C Mortality Score, respectively). The 4C Mortality Score and COVID-GRAM achieved the best performance in recognizing patients with WHO-defined severe or critical disease (AUC 0.785 and 0.717 for 4C Mortality Score and COVID-GRAM, respectively). However, neither of the prognostic indices discriminated well between patients requiring intensive care unit treatment or mechanical ventilation. In this context, the WHO severity classification on presentation achieved the highest, although modest, AUC values (AUC 0.667 and 0.687 for intensive care unit and mechanical ventilation, respectively) (Table 2).

**Table 2:** Prognostic properties of COVID-19 prognostic scores regarding different clinical outcomes, entire cohort

	30-day mortality	In-hospital mortality	World Health Organization (WHO) severe or critical disease	Intensive care unit	Mechanical ventilation
**The WHO COVID-19 severity classification categories**	0.672 (0.657-0.686)	0.675 (0.660-0.690)	-	0.667 (0.652-0.681)	0.687 (0.672-0.701)
**COVID-GRAM categories**	0.621 (0.602-0.640)	0.622 (0.603-0.641)	0.717 (0.699-0.734)	0.577 (0.558-0.597)	0.592 (0.576-0.615)
**The Veterans Health Administration COVID-19 (*VACO*) index categories**	0.699 (0.685-0.714)	0.695 (0.680-0.709)	0.603 (0.587-0.618)	0.545 (0.530-0.561)	0.569 (0.553-0.584)
**4C Mortality Score categories**	0.761 (0.748-0.775)	0.762 (0.748-0.775)	0.785 (0.772-0.798)	0.586 (0.571-0.602)	0.619 (0.604-0.634)
**CURB-65 categories**	0.761 (0.747-0.774)	0.757 (0.743-0.770)	0.684 (0.669-0.699)	0.617 (0.601-0.632)	0.649 (0.634-0.664)

*Data are presented as areas under the curve values with 95% confidence intervals.

### Comparison of predictive properties of COVID-19 prognostic scores in subgroups of patients with various disease severity

We further evaluated the performance of different prognostic scores in subgroups of patients with WHO-defined mild or moderate (Supplementary Table 1), severe (Supplementary Table 2), and critical intensity of symptoms at hospital admission (Supplementary Table 3). Patterns of performance of different prognostic scores in specific subgroups resembled those in the entire cohort. The CURB-65 and 4C Mortality scores performed substantially better regarding mortality prediction among patients with mild or moderate than among patients with severe or critical disease. The CURB-65 AUC values were 0.839, 0.707, and 0.769 for 30-day mortality and 0.825, 0.703, and 0.765 for in-hospital mortality in patients with mild or moderate, severe, and critical disease, respectively. Similarly, 4C Mortality Score AUC values were 0.842, 0.708, and 0.742 for 30-day mortality and 0.823, 0.707, and 0.748 for in-hospital mortality in patients with mild or moderate, severe, and critical disease, respectively.

### Independent prognostic properties of different prognostic scores

We analyzed all the investigated prognostic indices stratified by their respective prognostic categories in the Cox regression model for 30-day mortality (Table 3). WHO severe vs mild disease, WHO critical vs mild disease, COVID-GRAM high vs medium plus low risk, 4C Mortality Score very high vs low risk, and all prognostic CURB-65 categories remained significantly associated with a worse survival and performed independently of each other in distinguishing 30-day mortality. Prognostic properties of the VACO Index and lower-risk 4C Mortality Score categories were redundant as their prognostic categories remained insignificantly associated with survival when controlling for other scores.

**Table 3:** Assessment of mutually independent contribution of particular categories of individual prognostic scores for 30-day mortality prediction by using the Cox regression analysis*

Variable	P	Hazard ratio with 95% confidence interval
**WHO COVID-19 severity classification**		
moderate vs mild symptoms	0.621	0.7 (0.17-2.93)
severe vs mild symptoms	<0.001	6.71 (2.74-16.43)
critical vs mild symptoms	<0.001	9.11 (3.67-22.65)
**COVID-GRAM**		
high vs medium plus low risk	<0.001	2.59 (1.71-3.93)
**VACO index**		
moderate vs lower risk	0.151	0.78 (0.56-1.09)
high vs lower risk	0.877	1.03 (0.73-1.44)
extreme vs lower risk	0.373	1.16 (0.83-1.62)
**4C mortality**		
intermediate vs low risk	0.300	1.88 (0.57-6.21)
high vs low risk	0.107	2.76 (0.8-9.47)
very high vs low risk	0.020	4.4 (1.26-15.35)
**CURB-65**		
low vs very low risk	0.012	1.98 (1.16-3.38)
intermediate vs very low risk	<0.001	2.78 (1.58-4.87)
severe vs very low risk	<0.001	4.48 (2.5-8.05)
very severe vs low risk	<0.001	6.35 (3.39-11.86)

*Abbreviations: WHO – World Health Organization; VACO – The Veterans Health Administration COVID-19 Index.

## Discussion

In the current study, all of the investigated prognostic models were able to identify groups of patients with a worse prognosis. However, the scores performed differently in terms of prediction of clinical outcomes of interest, as well as in terms of the number of prognostic categories they were able to distinguish.

Specific COVID-19 scores did not outperform the classical CURB-65 score, developed for community-acquired pneumonia. Also, the predictive properties of specific scores were mostly lower than in the original patient cohorts or other validation studies (10,18), a finding that further highlights the differences among various clinical contexts and the importance of real-life data. The best predictive properties were observed among patients with mild or moderate disease symptoms. The CURB-65 and 4C Mortality Score performed best at correctly recognizing patients with inferior 30-day mortality and in-hospital mortality. Both scores include information on age, respiratory, hydration, and mental status, with the 4C Mortality Score additionally including information on comorbidity burden and inflammation. These two scores comparably distinguished between survival-related outcomes despite the lower number of variables and no information on patient history required for the calculation of the CURB-65 score. The CURB-65 also provides the highest number of prognostic categories (five) compared with all other scores, enabling more precise risk stratification.

The investigated prognostic scores provided additional prognostic information regarding 30-day mortality one to another, with the exception of the VACO Index, whose prognostic properties were redundant when evaluated synchronously with other prognostic scores. The VACO Index is based on age, sex, and the number of comorbidities but does not provide information on the current inflammatory and respiratory status. Its strength is the prediction of the risk associated with future COVID-19 infection, but it may not perform as well as other scores at hospital admission either regarding the prediction of particular outcomes or regarding additional prognostic information. The VACO Index may be improved by adding parameters reflecting the acute state of the patient (19). Since many COVID-19 prognostic scores include information on comorbidities, their use requires profound knowledge on the patient’s history, which may not be available in the pandemic working conditions, where incomplete medical records and patients’ inability to provide correct history due to confusion are common. Simple and quick-to-obtain biochemical parameters such as red cell distribution width (RDW) and CRP-to-albumin ratio (CAR) provide additional prognostic information to COVID-19 prognostic scores (9,20). Since they are non-specific and may be profoundly affected by comorbidities (like RDW) or COVID-19 associated inflammation (CAR), they represent excellent candidates to be added to the current prognostic scores and to allow more precise risk stratification.

Limitations of the study are the single-center experience, retrospective design, and study period before or at the very beginning of the vaccination program. Our data are representative of a tertiary referral center with a high number of mostly elderly, severe or critical COVID-19 patients with a number of acute or chronic medical conditions. Thus, our results provide a unique overview specific to this clinical context. Our results need confirmation from studies on independent data sets.

In conclusion, complex prognostic scores based on a large number of parameters and comorbid conditions did not achieve better prognostic properties for survival outcomes of hospitalized COVID-19 patients in comparison with a simple CURB-65 prognostic score. The CURB-65 also provides the largest number of prognostic categories (five), allowing more precise risk stratification than other prognostic scores.
